# GPC1 Is Associated with Poor Prognosis and Treg Infiltration in Colon Adenocarcinoma

**DOI:** 10.1155/2022/8209700

**Published:** 2022-09-14

**Authors:** Ying Liu, Hui Ren, Mu-qing Yang, Ji-yu Li

**Affiliations:** ^1^Department of General Surgery, Shanghai Tenth People's Hospital, School of Medicine, Tongji University, Shanghai 200092, China; ^2^School of Pharmacy, East China University of Science and Technology, Shanghai, China; ^3^Geriatric Cancer Center, Huadong Hospital Affiliated to Fudan University, Shanghai 200040, China

## Abstract

Glypican-1 (GPC1) is a glycosylated protein recognized as a promising biomarker for cancer. Nonetheless, there have been few systematic studies on GPC1 in colon adenocarcinoma (COAD). We conducted bioinformatic analysis based on The Cancer Genome Atlas (TCGA) and used clinical samples to verify that GPC1 is overexpressed in colon adenocarcinoma. Kaplan-Meier analysis showed that higher GPC1 expression was associated with poor overall survival (OS). The Cox regression model further showed that GPC1 expression is an independent negative prognostic factor for COAD. Gene set enrichment analysis demonstrated that multiple oncogenic signaling pathways were differentially enriched in GPC1 high- versus low-expressing COAD tumors, including DNA methylation, G2/M damage checkpoint, and telomere dysfunction. We observed a positive correlation between GPC1 expression and immune cell infiltration, such as regulatory T cells (Tregs), macrophages, and mast cells, and immunohistochemistry of 50 COAD tissues revealed that GPC1 expression was positively associated with Treg enrichment. Our results provide a promising candidate gene to predict the prognosis of COAD and new insights into tumor immunity. Further research is required to validate these results.

## 1. Introduction

Colorectal cancer is the third most common cancer worldwide, accounting for 1.4 million new cases annually [1]. Most colon cancers are sporadic in nature. Western diets, chronic inflammation, environmental factors, and colonic polyps have been implicated in their pathogenesis. The most common type of colon cancer is colon adenocarcinoma (COAD), which accounts for more than 90% of colon cancers. In recent years, with the development of living standards and changes in diet, the incidence of colorectal cancer has increased. Despite advances in screening, surgery, and treatment, colorectal cancer remains the third leading cause of cancer-related deaths worldwide, with nearly 900,000 deaths in 2018 [2]. These observations underscore the need to identify new biological markers.

Glypican-1 (GPC1) is one of six members of the glycosaminoglycan-anchored cell surface glypican protein family [3, 4]. Glypicans are predominantly expressed during embryonic development and have been reported to play an important role in organ morphological development by influencing signaling pathways including Wnt, hedgehog, transforming growth factor-*β*, and fibroblast growth factor [5, 6]. Glypicans participate in many important processes, including cellular proliferation, migration, differentiation, extracellular matrix, and tumor microenvironment remodeling. Studies have suggested that aberrant expression of GPC1 is detected in multiple cancers and that disturbance of GPC1 influences cancer progression. GPC1 has been proven to be a useful biomarker for multiple cancerous tissues, such as prostate, hepatocellular, and pancreatic carcinomas [7–9]. A mouse model of sporadic colorectal cancer showed that mouse adenocarcinoma tissues contained much higher levels of GPC1 than normal tissues [10]. Papiewska-Pajak et al. demonstrated that exosomes released from the mouse colon adenocarcinoma cell line MC38, after stimulation by the transcription factor Snail, presented higher levels of GPC1 [11]. Nonetheless, there are few systematic studies of GPC1 on the progression, prognosis, and immune response in COAD using bioinformatic approaches, especially in human colorectal cancer samples. This study is aimed at exploring the potential biological value of GPC1 in prognosis and immunity, thereby providing new insights into COAD.

## 2. Materials and Methods

### 2.1. Data Source and Comparison of GPC1 Expression

The Fragments Per Kilobase per Million (FPKM) RNAseq dataset from TCGA, containing 480 tumor samples, 41 normal samples, and their general and clinical data, was downloaded for expression analysis (https://portal.gdc.cancer.gov/). The tumor samples included 41 matched COAD and normal adjacent tissues. The transcript data were converted to transcripts per million (TPM) format data before analysis. The R pROC package was used for receiver operating characteristic (ROC) curve analysis.

The Ethics Committee of Shanghai Tenth People's Hospital (no. 20KT95) approved the use of tissue microarray slides (Biotech, D100Co01, Xi'an, China) with 50 matched pairs of COAD samples and adjacent normal tissues for immunohistochemical (IHC) analysis. All patients were pathologically diagnosed with COAD, and none of them had received preoperative therapy or had a history of other malignant tumors. Basic clinical information is summarized in Table S1.

### 2.2. Correlation between GPC1 Expression and Clinicopathological Parameters and Prognosis

Correlation analyses were performed with R statistical software using the “ggplot2” package. The patients were divided into two groups based on the median: high (above the median) and low (below the median). Disparities in survival by GPC1 expression and Kaplan-Meier (KM) curves were performed using the R package “survival” and “survminer.”

### 2.3. Univariate and Multivariate Cox Hazard Regression Analyses

Cox proportional hazard model analyses were generated using the R packages “survminer” and “survival.” In the univariate Cox hazard regression analysis, age, sex, race, T stage, N stage, M stage, pathologic stage, and GPC1 expression were set as independent variables, and overall survival (OS) was the dependent variable. A univariate Cox regression analysis was performed for each prognostic variable. To identify independent prognostic factors, all significant variables (*p* < 0.1) on univariate Cox regression analysis were further evaluated using multivariate Cox regression analysis.

### 2.4. Construction and Assessment of GPC1-Based Nomogram

Based on the results of the Cox hazard regression analysis, a nomogram was established using the rms package in R to analyze and visualize the multiple risk factors for predicting OS. Calibration plots were generated using the “rms” R package to show the predicted probabilities versus actual probabilities at multiple time points.

### 2.5. GPC1-Related Signaling Pathways according to Gene Set Enrichment Analysis (GSEA)

Differential expression between the high and low groups based on the median was determined using the R package DESeq2. Using this procedure, we obtained the logFC and *p* value for each gene. GSEA was performed using the Cluster Profiler R package and the MSigDB collections. Only pathways with FDR *q* values < 0.25, *p*.adjust < 0.05, and |NES| > 1 were considered significantly enriched.

### 2.6. Correlation between GPC1 and Immunity

Single-sample GSEA was performed in the R package GSVA to analyze immune cell enrichment in the two groups. The R package was used to calculate the immune, stromal, and estimate scores.

### 2.7. IHC

Primary antibodies included anti-GPC1 (Proteintech, Rosemont, IL, USA) and anti-Foxp3 (Proteintech, Rosemont, IL). Three microscopic fields (magnification, ×40) were randomly selected for analysis. The IHC score of GPC1 was calculated by multiplying the intensity of IHC staining (intensity score: 1-4) by the percentage of positive cells (percentage score: 1-4). The number of Foxp3-positive cells was counted under a high-power microscope. The mean values were used as the final results.

### 2.8. Statistical Analysis

Statistical analyses were performed using R statistical software (version 3.6.3). When normality and homogeneity of variance were met, Student's *t*-test was used to analyze the differences between the two groups. The Wilcoxon rank-sum test was used when the variance did not satisfy the normality or homogeneity. Differences between two matched datasets were analyzed using paired Student's *t*-test. Analysis of multiple groups was performed using the Kruskal-Wallis test. The influence of prognostic factors on survival was evaluated using the Cox proportional hazard regression model. Correlations were tested using Pearson's method. If normality assumptions were not met, Spearman's method was used. Correlations between GPC1 expression and immune cell infiltration, immune score, stromal score, and estimated score were assessed using the Spearman analysis. For all data, ^∗^*p* < 0.05,  ^∗∗^*p* < 0.01,  ^∗∗∗^*p* < 0.001, and^∗∗∗∗^*p* < 0.0001; ns means not significant.

## 3. Results

### 3.1. GPC1 Expression Is Elevated in COAD

**Figure 1:**
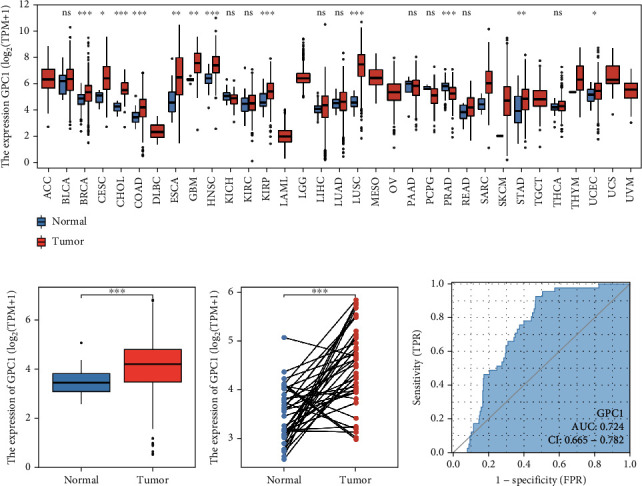
GPC1 expression is increased in COAD: (a) GPC1 mRNA expression of different cancers compared with corresponding normal tissues in TCGA database; (b, c) GPC1 mRNA expression in COAD and normal tissue (unpaired tissues (b) and paired tissues (c)) in TCGA database; (d) ROC curve to test the value of GPC1 to identify COAD tissues.

Based on TCGA data, we found that GPC1 expression was upregulated in multiple cancers, including breast cancer, cervical cancer, bile duct cancer, colon cancer, glioblastoma, head and neck cancer, kidney papillary cell carcinoma, lung adenocarcinoma, and pancreatic cancer (Figure 1(a)). Next, we focused on COAD data from TCGA for further analysis. Compared to normal tissues, GPC1 mRNA was expressed at higher levels in COAD tissues (Figure 1(b), *N* = 41, *T* = 41, *p* < 0.001). We screened 41 paired COAD and normal tissues from TCGA data. Similarly, GPC1 expression was upregulated in tumor tissues (Figure 1(c), *p* < 0.001). The area under the ROC curve (AUC) was 0.724 (Figure 1(d)), indicating that GPC1 may potentially be able to distinguish COAD from normal tissue.

### 3.2. GPC1 Expression Correlates with Clinicopathological Characteristics and Poor Prognosis

**Figure 2:**
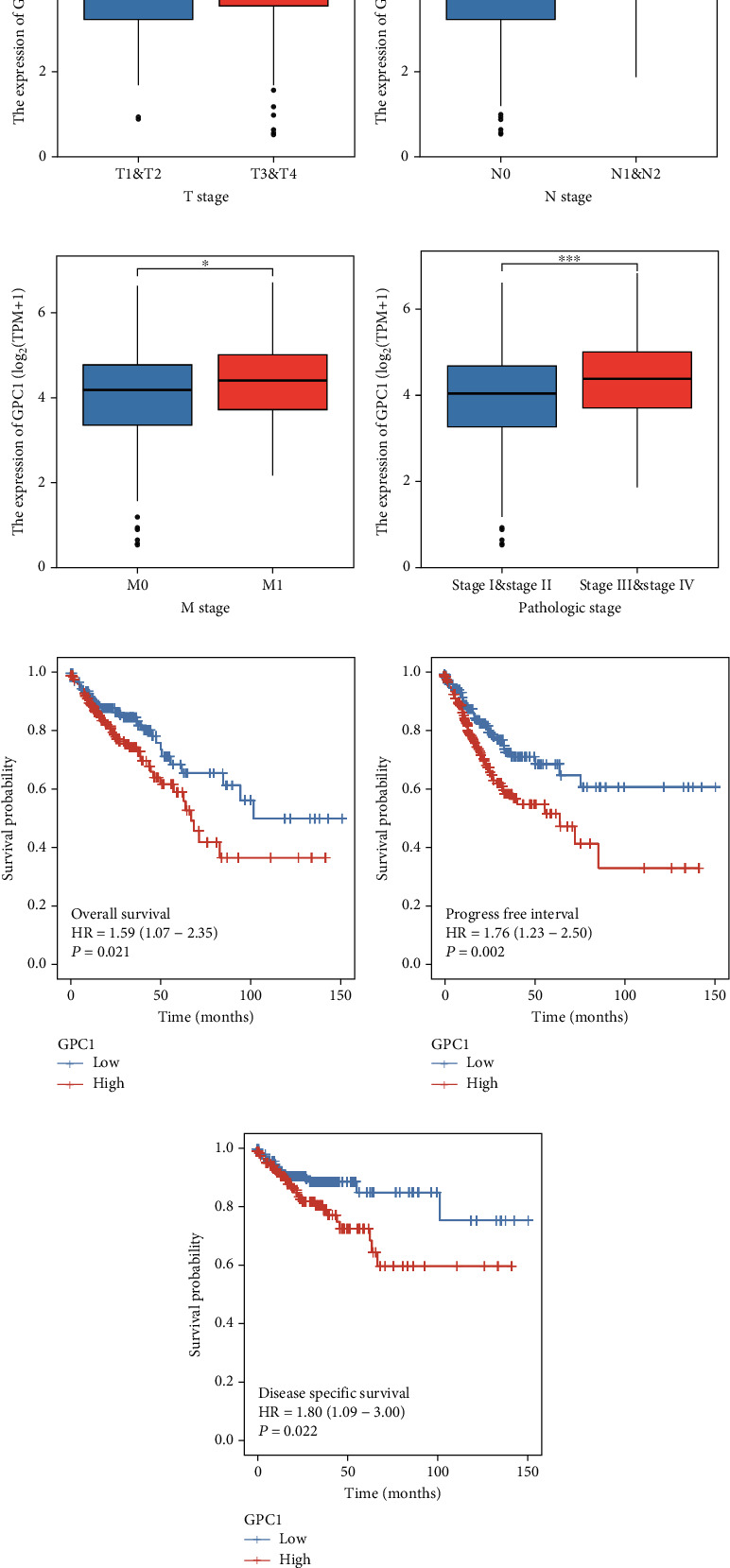
Associations between GPC1 expression and clinicopathological features and prognosis: (a–d) associations between GPC1 expression and T stage (a), N stage (b), M stage (c), and pathologic stage (d); (e–g) Kaplan-Meier analysis of OS (e), progress-free interval (f), and disease-specific survival (g) in COAD in TCGA database.

Next, we evaluated whether GPC1 expression is associated with clinicopathological characteristics. We found that high GPC1 expression was associated with advanced T stage (Figure 2(a), T1&T2 = 94, T3&T4 = 383; *p* = 0.038), N stage (Figure 2(b), N0 = 284, N1&N2 = 194, *p* < 0.001), M stage (Figure 2(c), M0 = 349; M1 = 66; *p* = 0.04), and pathologic stage (Figure 2(d), Stage I and Stage II = 268, Stage III and Stage IV = 199, *p* < 0.001), indicating that patients with more advanced tumor stage or higher grade tumors were more likely to have higher levels of GPC1. Next, we analyzed the correlation between GPC1 expression and prognosis using TCGA data. KM analyses revealed that patients with low GPC1 expression had longer OS (Figure 2(e), *p* = 0.021, HR = 1.59), progression-free interval (Figure 2(g), *p* = 0.002, HR = 1.76), and disease-specific survival (Figure 2(f), *p* = 0.022, HR = 1.80) than those with high expression. Two groups were divided based on the median (high = 239, low = 238).

Univariable and multivariable Cox proportional hazard regression analyses were performed to identify potential factors associated with the OS of patients with COAD. In univariable Cox proportional hazard regression analysis, OS was associated with age (≤65 = 194, >65 = 283, *p* = 0.028, HR = 1.610), T stage (T1&T2 = 94, T3&T4 = 382, *p* = 0.004, HR = 3.072), N stage (N0 = 283, N1&N2 = 194, *p* < 0.001, HR = 2.592), M stage (M0 = 348, M1 = 66, *p* < 0.001, HR = 4.193), pathologic stage (Stage I and Stage II = 267, Stage III & Stage IV = 199, *p* < 0.001, HR = 2.947), and GPC1 (log2(TPM + 1), *p* = 0.002, HR = 1.339). Variables significant in the univariate Cox regression analysis (*p* < 0.1) were subjected to further multivariate Cox regression analysis. The results showed that OS was associated with age (*p* < 0.001, HR = 2.553), T stage (*p* = 0.039, HR = 3.471), M stage (*p* = 0.003, HR = 2.264), pathological stage (*p* = 0.007, HR = 1.225), and GPC1 (*p* = 0.048, HR = 1.225). Overall, age, T stage, M stage, pathological stage, and GPC1 expression were independent prognostic factors for patients with COAD (Table 1).

### 3.3. Establish and Evaluate the Prognostic Models Based on GPC1

**Figure 3:**
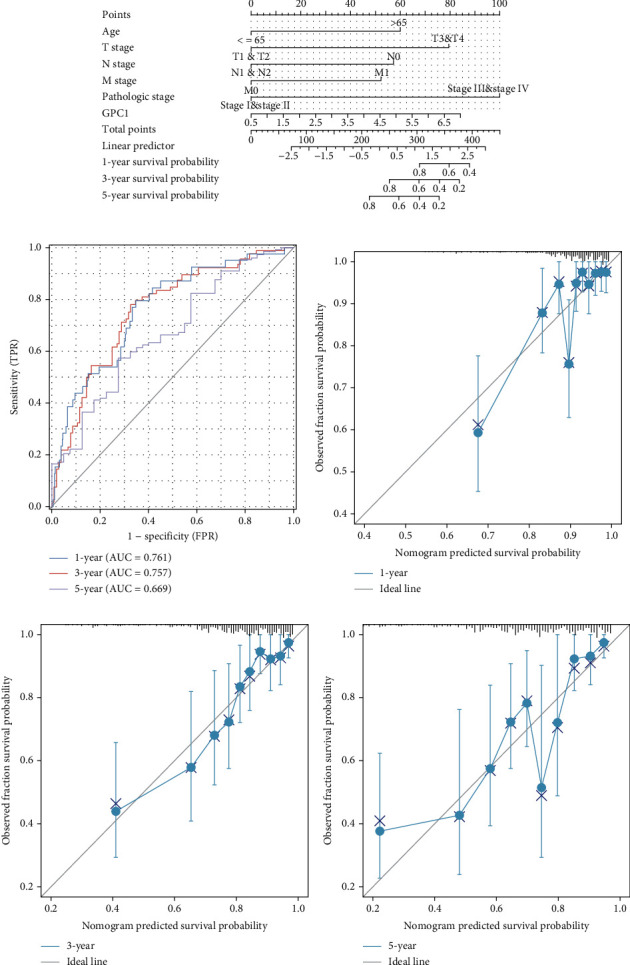
Prognostic value of the mRNA level of GPC1 in patients with COAD: (a) nomogram based on variables including gender; T, N, and M stage; and GPC1 expression in COAD from TCGA datasets; (b) 1-, 3-, and 5-year ROC curve; (c–e) 1-, 3-, and 5-year calibration curve.

Based on the independent factors identified in the Cox regression analyses, we created a nomogram for COAD patients to show the associations with OS of 1, 3, and 5 years (Figure 3(a)). ROC analysis was used to quantify the accuracy of the prognostic model. The AUCs at 1 year, 3 years, and 5 years were 0.761, 0.757, and 0.669, respectively (Figure 3(b)), indicating moderate predictive power for 1 year and 3 years. The c-index for the multivariable model was 0.758, which also implies moderate predictive power. Moreover, the calibration plot was close to the diagonal, confirming that they were close to the ideal (Figures 3(c)–3(e)).

### 3.4. GPC1-Related Signaling Pathways according to GSEA

**Figure 4:**
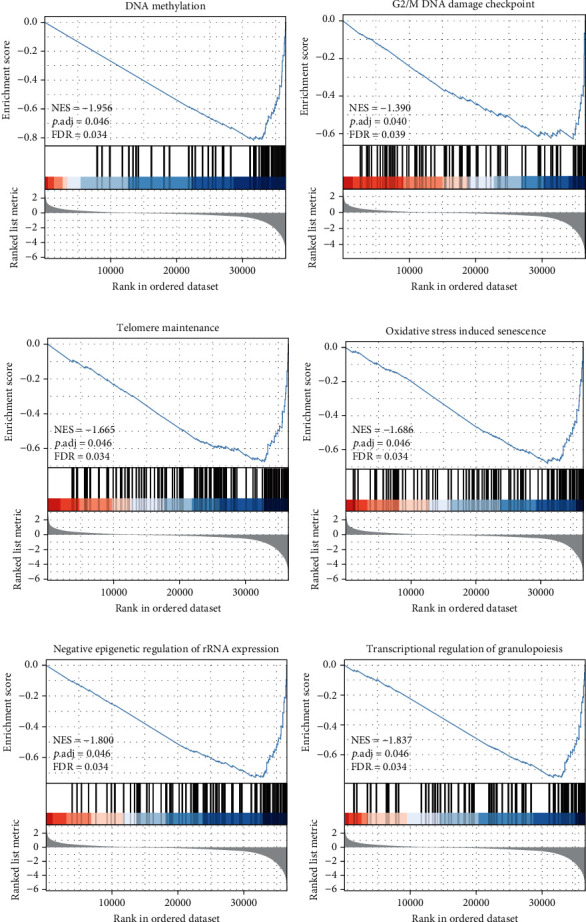
GSEA results based on GPC1 mRNA expression in COAD from TCGA datasets. (a–f) Significantly enriched pathway of DNA methylation, G2/M DNA damage checkpoint, telomere maintenance, oxidative stress-induced senescence, negative epigenetic regulation of rRNA expression, and transcriptional regulation of granulopoiesis.

To explore the biological functions of GPC1, we investigate GPC1-associated signaling pathways enriched differently between GPC1-high and GPC1-low patients by GSEA. We found that several pathways showed significant enrichment, such as DNA methylation, G2/M DNA damage checkpoint, telomere maintenance, oxidative stress-induced senescence, negative epigenetic regulation of rRNA expression, and transcriptional regulation of granulopoiesis signaling pathways (Figures 4(a)–4(f)).

### 3.5. GPC1 Is Related to Immune Cell Infiltration in COAD

**Figure 5:**
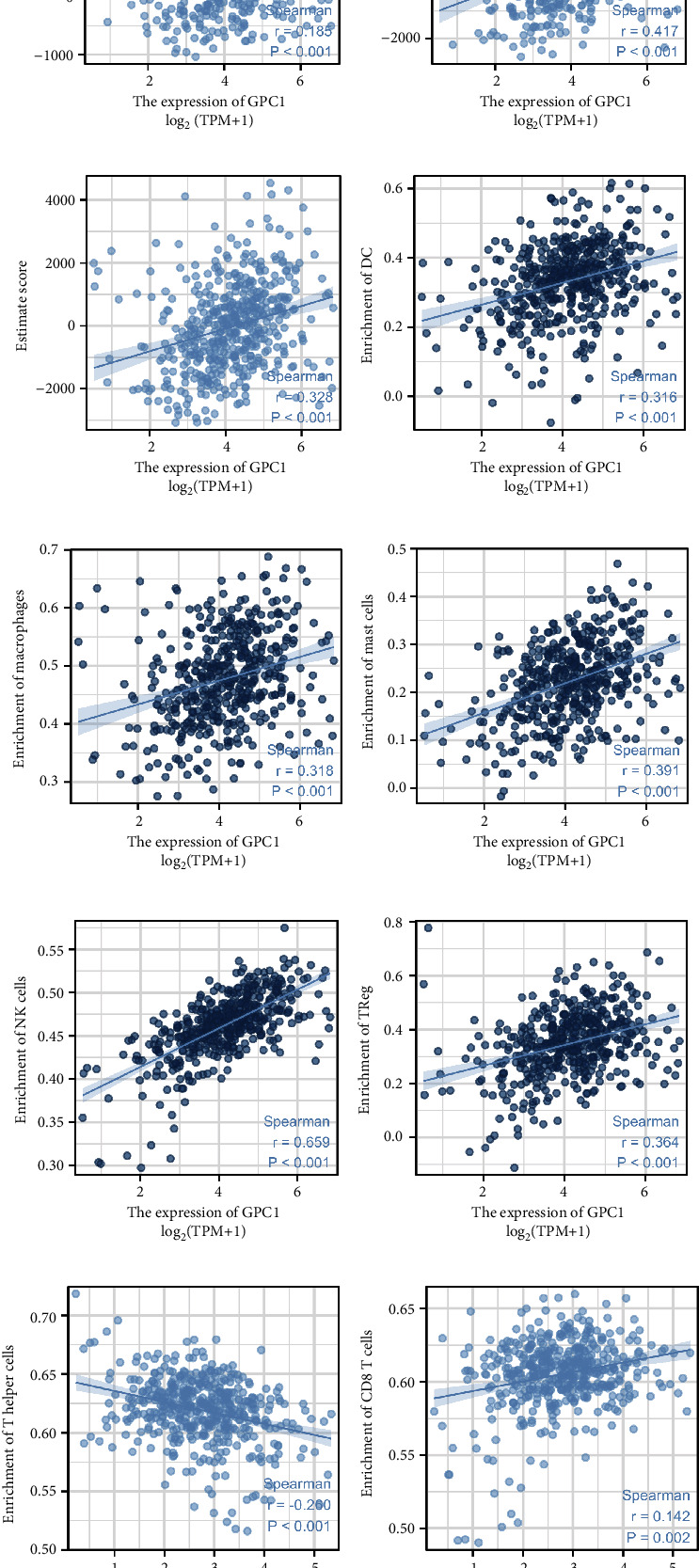
GPC1 is associated with proportions of immune cells: (a) immune cell distribution in patients with high GPC1 expression level and low GPC1 expression level; (b–d) associations between the expression of GPC1 and the immune score, stromal score, and estimate score; (e–l) associations between the expression of GPC1 and enrichment of immune cell (DC, macrophage, mast cells, NK cells, Tregs, T helper cells, CD8 T cells, and neutrophils).

The tumor immune microenvironment and its various cellular components play a crucial role in cancer progression. Thus, we explored the correlation between GPC1 expression and tumor immunity based on TCGA data. COAD with high GPC1 expression presented higher infiltration levels of B cells, CD8+T cells, cytotoxic cells, dendritic cells (DC), eosinophils, macrophages, mast cells, neutrophils, natural killer (NK) cells, effective memory T (Tem) cells, TH1 cells, and Treg cells and lower infiltration levels of T helper cells and Th2 cells (Figure 5(a)). Spearman correlation revealed that the expression of GPC1 was positively correlated with the immune score (Figure 5(b), *r* = 0.185, *p* < 0.001), stromal score (Figure 5(c), *r* = 0.417, *p* < 0.001), and estimated score (Figure 5(d), *r* = 0.328, *p* < 0.001). In other words, the higher the level of GPC1, the higher the degree of immune and stromal infiltration. Moreover, GPC1 expression was positively associated with the enrichment of DC (Figure 5(e), *r* = 0.316, *p* < 0.001), macrophages (Figure 5(f), *r* = 0.318, *p* < 0.001), mast cells (Figure 5(g), *r* = 0.391, *p* < 0.001), NK cells (Figure 5(h), *r* = 0.659, *p* < 0.001), and Treg cells (Figure 5(i), *r* = 0.364, *p* < 0.001). However, no significant relationship was found between GPC1 expression and the enrichment of T helper cells (Figure 5(g), *r* = 0.260, *p* < 0.001), CD8 T cells (Figure 5(k), *r* = 0.142, *p* = 0.002), and neutrophils (Figure 5(l), *r* = 0.188, *p* < 0.001).

### 3.6. Validation Using Clinical Specimens

**Figure 6:**
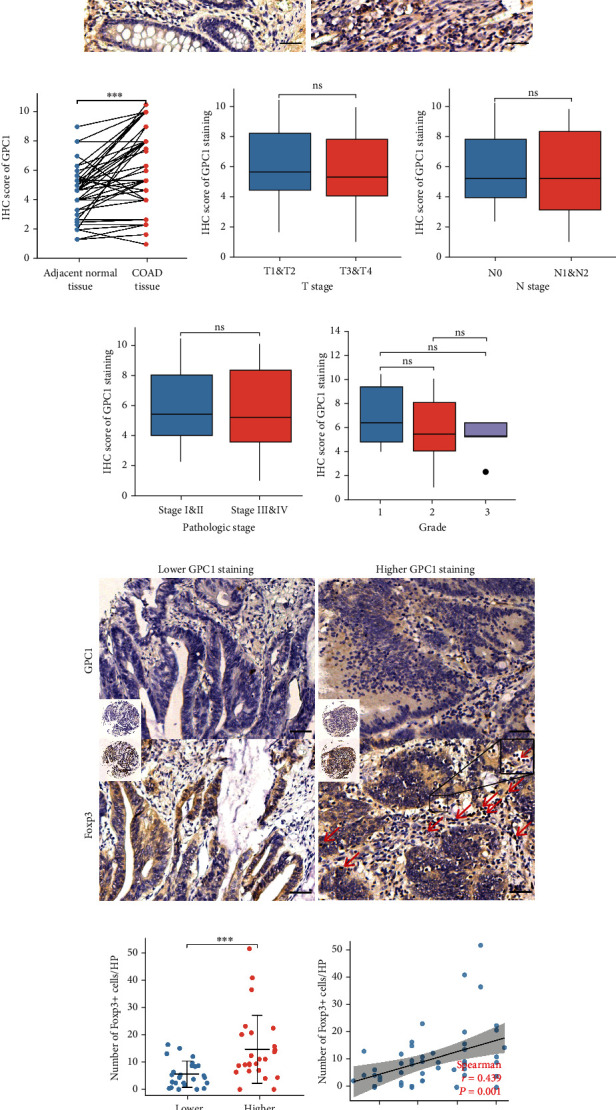
Validation using clinical specimens: (a) representative images of IHC staining of GPC1 in COAD and paired adjacent normal tissues; (b) IHC score of GPC1 of COAD compared with corresponding adjacent normal tissues; (c–f) associations between GPC1 IHC score and T stage ((c) T1&T2 = 12, T3&T4 = 38; *p* = 0.638), N stage ((d) N0 = 35, N1&N2 = 15, *p* = 0.782), pathologic stage ((e) Stage I & Stage II = 37, Stage III & Stage IV = 16; *p* = 0.961), and pathologic grade ((f) Grade 1 = 6, Grade 2 = 39, and Grade 3 = 5; *p* = 0.892); (g) representative images of IHC staining of GPC1 and Foxp3 in COAD; (h) number of Foxp3+ cells per high power field (HP) in GPC1 high- versus low-expressing groups; (i) associations between GPC1 IHC score and number of Foxp3+ cells per HP. Black scale bar = 50 *μ*m. Red arrow points to Foxp3+ cells.

IHC was performed on 50 matched pairs of COAD and normal tissue specimens. Representative IHC staining images of GPC1 are shown in Figure 6(a). COAD exhibited an elevated IHC score for GPC1 compared to matched normal tissues (Figure 6(b), *n* = 50, *p* < 0.001). Next, we analyzed the correlation between the clinical characteristics of patients and the IHC score of GPC1. Unfortunately, no correlation was observed in our specimens (Figures 6(c)–6(f)). We also performed IHC staining for Foxp3 in all tumor samples to label Tregs. GPC1 and Foxp3 staining was conducted on serial sections, and a schematic diagram is depicted in Figure 6(g). The patients were divided into the low- and high-expression groups based on the median IHC score. The comparison indicated that higher GPC1 expression tended to be associated with a higher frequency of Foxp3+ cells (Figure 6(h), *n* = 25, *p* = 0.001). Spearman's correlation analysis showed that the IHC score of GPC1 staining was positively associated with Foxp3+ cell frequency in COAD (Figure 6(i), *r* = 0.439, *p* = 0.001).

## 4. Discussion

Previous studies have demonstrated abnormal GPC1 expression in tumor tissues of pancreatic ductal adenocarcinoma and prostate cancer, but GPC1 was nearly absent in normal and adjacent tissues [12, 13]. In cancer tissues of esophageal squamous cell carcinomas, cervical cancers, and glioma, GPC1 expression levels were higher than those in normal tissues [14–16]. Other investigations have recently suggested that GPC1 expression is a prognostic biomarker for pancreatic ductal adenocarcinoma [12, 17]. As GPC1 also has a secreted soluble form, it has been reported to be detected in serum exosomes and is promising for the early detection of pancreatic cancer [18]. Because its plasma, serum, and urine levels are elevated in patients with prostate carcinoma, GPC1 might serve as a reliable diagnostic marker for prostate cancer [19, 20]. These results suggest that GPC1 has a potential clinical value as a predictive and prognostic biomarker for several cancers. However, the role of GPC1 in patients has rarely been studied. A previous study showed that plasma GPC1+ exosomes were significantly increased in colorectal cancer patients compared to healthy patients and reduced after surgery [21]. Notably, one disadvantage of serum is that its composition is source-dependent, and serum GPC1 may be confounded by other sources, as GPC1 is widely expressed in mammalian tissues. In addition, the release of GPC1 relies on protease notum [4], which has not been fully studied in normal and cancerous tissues. Concurrently, IHC analysis of 50 paired human tissues confirmed higher GPC1 protein expression in COAD tissues. These results suggest that GPC1 may be a potential clinical biomarker and exert active biological functions in COAD.

Based on TCGA data, we found that GPC1 was associated with the tumor stage. However, our external validation did not support this view. In univariate and multivariate Cox regression analyses, the GPC1 expression level was an independent negative prognostic factor. To validate our results, we established a nomogram and calibration models to predict the survival probabilities of patients with COAD. These results, including the ROC curve, showed that our model had a moderate ability to predict the prognosis.

GSEA was performed to explore differentially expressed genes and pathway enrichment in GPC1 high- versus low-expressing COAD tumors in TCGA dataset. Results showed that multiple oncogenic signaling pathways were found to be aberrantly expressed. DNA methylation is an epigenetic process that modulates gene expression. Aberrant DNA methylation is one of the earliest and most universal signatures of cancer [22, 23]. Multiple biomarker-based DNA methylations have been used in the prediction, diagnosis, and prediction of therapeutic approaches for colorectal cancer [24]. The G2/M damage checkpoint prevents cells from entering M phase and promotes DNA damage repair processes, which is important for malignant transformation [25]. Chromosome instability triggered by telomere dysfunction is considered a promoter of tumorigenesis. Studies have revealed that telomeres in human colon cancer are often shorter than those in normal tissues, which is a key driving event in colorectal carcinomas [26, 27].

The composition of infiltrating immune cells influences the immune status of the tumor microenvironment. Tumors evade the immune system by establishing an immunosuppressive tumor microenvironment. Tregs are a predominant subset of suppressor T cells that play a vital role in tumor tolerance. Tregs exert immunosuppressive functions through the secretion of inflammatory cytokines such as IL-10, IL-35, and TGF-*β*, inducing target immune cell death by granzyme B and negatively regulating DC maturation and self-antigen presentation via negative costimulatory molecules [28–30]. Although the prognostic value of Foxp3+ Tregs in COAD is controversial [31], some studies have shown that the role of Treg cells in the tumor microenvironment is unclear and that Tregs may play a role in promoting antitumor immunity. Research on colorectal cancer has demonstrated that increased frequencies of Treg cells are often correlated with tumor immune evasion and poor prognosis of patients [32]. Because we found that higher GPC1 expression indicated a worse prognosis in COAD patients, we speculated that the frequency of intratumoral Treg cells correlated positively with GPC1 expression. However, we have only demonstrated the correlation between the two and have not demonstrated that GPC1 has a chemotactic effect on Treg cells or could regulate their proliferation. Previous research has shown that TGF-*β* signaling is fundamental for both Th17 and Treg cell differentiation. GPC1 modulates various signaling pathways, including TGF-*β*. In the future, we hope to explore whether GPC1 directly affects the infiltration of Treg cells and its mechanism. Our findings on the potential association between GPC1 and Tregs provide direction for future COAD studies.

Some of the data analyzed in this study were derived from public databases, and the present study has some limitations. Further research is needed to validate our findings and investigate the biological functions of GPC1 in COAD.

## 5. Conclusions

In this study, we addressed the role of GPC1 in prognosis and immune cell infiltration in colon cancer for the first time based on TCGA datasets. GPC1 is overexpressed in colon adenocarcinoma and is associated with poor OS. GPC1 expression is an independent negative prognostic factor for COAD, and DNA methylation, the G2/M damage checkpoint, and telomere dysfunction were differentially enriched in GPC1 high- versus low-expressing COAD tumors. In addition, we observed a positive correlation between GPC1 expression and immune cell infiltration based on TCGA dataset. The IHC results of clinical samples revealed that GPC1 expression was positively associated with the enrichment of Tregs. However, further in vivo and in vitro evidence is needed to elucidate that GPC1 may participate in immune cell infiltration and influence prognosis.

## Figures and Tables

**Table 1:** Univariate and multivariate Cox hazard regression analyses of COAD in TCGA.

Variables	Total (*n* vs. *n*)	Univariate analysis		Multivariate analysis	
		HR (95% CI)	*p* value	HR (95% CI)	*p* value
Age (>60 vs. ≤60)	477 (194 vs. 283)	1.610 (1.052-2.463)	0.028	2.553 (1.538-4.241)	<0.001
Gender (female vs. male)	477 (226 vs. 251)	1.101 (0.746-1.625)	0.627		
Race (Asian vs. Black or African-American vs. White)	306 (11 vs. 63 vs. 232)	0.927 (0.208-4.133)	0.921		
T stage (T1&T2 vs. T3&T4)	476 (94 vs. 382)	3.072 (1.423-6.631)	0.004	3.471 (1.066-11.305)	0.039
N stage (N0 vs. N1&N2)	477 (283 vs. 194)	2.592 (1.743-3.855)	<0.001	0.409 (0.152-1.097)	0.076
M stage (M0 vs. M1)	414 (348 vs. 66)	4.193 (2.683-6.554)	<0.001	2.264 (1.309-3.915)	0.003
Pathologic stage (I&II vs. III&IV)	466 (267 vs. 199)	2.947 (1.942-4.471)	<0.001	4.772 (1.528-14.900)	0.007
GPC1 (log_2_(TPM + 1))	477	1.339 (1.114-1.609)	0.002	1.225 (1.002-1.497)	0.048

## Data Availability

The data that support the findings of this study are available from the corresponding author upon reasonable request.

## Supplementary Materials

Supplementary Materials See Table S1 in the Supplementary Materials for comprehensive analysis.
