# Supplementary material for: GPC1 Is Associated with Poor Prognosis and Treg Infiltration in Colon Adenocarcinoma
Source: Comput Math Methods Med. 2022 Sep 14;2022:8209700. doi: 10.1155/2022/8209700 (PMC9492339; doi:10.1155/2022/8209700)
Supplement: Supplementary Materials — See Table S1 in the Supplementary Materials for comprehensive analysis. [file 8209700.f1.docx]

| Characteristic | TCGA | Clinical samples |
| --- | --- | --- |
| n |  |  |
| Age, n (%) |  |  |
| <=65 |  |  |
| >65 |  |  |
| Gender, n (%) |  |  |
| Female |  |  |
| Male |  |  |
| Race |  |  |
| Asian |  |  |
| Black or African American |  |  |
| White |  |  |
| T stage, n (%) |  |  |
| T1 |  |  |
| T2 |  |  |
| T3 |  |  |
| T4 |  |  |
| N stage, n (%) |  |  |
| N0 |  |  |
| N1 |  |  |
| M stage, n (%) |  |  |
| M0 |  |  |
| M1 |  |  |
| Pathologic stage |  |  |
| Ⅰ |  |  |
| Ⅱ |  |  |
| Ⅲ |  |  |
| Ⅳ |  |  |
